# Exploring the Probiotic Potential of *Bacteroides* spp. Within One Health Paradigm

**DOI:** 10.1007/s12602-024-10370-9

**Published:** 2024-10-08

**Authors:** Muhammad Aammar Tufail, Ruth A. Schmitz

**Affiliations:** Institut für Allgemeine Mikrobiologie, Christian-Albrechts-Universität zu Kiel, 24118 Kiel, Germany

**Keywords:** Probiotics, *Bacteroides* spp., Human health, Novel probiotics, One health

## Abstract

Probiotics are pivotal in maintaining or restoring the balance of human intestinal microbiota, a crucial factor in mitigating diseases and preserving the host’s health. Exploration into *Bacteroides* spp. reveals substantial promise in their development as next-generation probiotics due to their profound interaction with host immune cells and capability to regulate the microbiome’s metabolism by significantly impacting metabolite production. These beneficial bacteria exhibit potential in ameliorating various health issues such as intestinal disorders, cardiovascular diseases, behavioral disorders, and even cancer. Though it’s important to note that a high percentage of them are as well opportunistic pathogens, posing risks under certain conditions. Studies highlight their role in modifying immune responses and improving health conditions by regulating lymphocytes, controlling metabolism, and preventing inflammation and cancer. The safety and efficacy of *Bacteroides* strains are currently under scrutiny by the European Commission for authorization in food processing, marking a significant step towards their commercialization. The recent advancements in bacterial isolation and sequencing methodologies, coupled with the integration of Metagenome-Assembled Genomes (MAGs) binning from metagenomics data, continue to unveil the potential of *Bacteroides* spp., aiding in the broader understanding and application of these novel probiotics in health and disease management.

## Introduction

The human gut microbiota refers to the collection of microorganisms in the gut, which includes bacteria, archaea, and eukarya (like fungi) and phages/viruses, that colonize the gut from birth and undergo changes throughout life in response to dietary factors, health conditions, and aging [58]. The number of these important symbionts fluctuates depending on where they colonize [39]. Besides degradation of organic material generating energy, they have a significant impact on health and well-being, influencing the immune system, metabolism, detoxification processes, and even behavior [35, 111]. Maintaining a healthy gut microbiota is crucial for overall health, as changes in its composition have been associated with different life phases, diet, exercise, antibiotic use, and various disease states [4, 58, 109]. Any disturbance in the gut microbiota including low bacterial diversity, decrease in beneficial microbial products, and accumulation of virulent agents can cause several negative effects such as, inflammation, frailty, and impaired cognitive function [27]. Therefore, a balanced gut microbiome with high diversity through nutrition, stress management, and medicinal interventions is crucial for overall health and well-being. Furthermore, probiotics, prebiotics, and antibiotics are thought to be the most effective treatments for the conditions caused by disturbance in the gut microbiota [31, 58, 64]. Therefore, understanding the interactions between the gut microbiota and host health is essential for managing and potentially manipulating it for therapeutic purposes [176].

Prebiotics are defined as non-digestible food ingredients that promote the growth or activity of beneficial microorganisms already present in the colon by serving as fuel for probiotics and stimulating their growth and activity, with examples including dietary fibers such as inulin and fructooligosaccharides [55, 158]. Probiotics are defined as live microorganisms, such as *Lactobacillus, Bifidobacterium*, and *Escherichia coli* (Nissle 1917 strain) that when administered in adequate amounts and duration of administration confer health benefits to the host by modulating the gut microbiota and mucosal immune system through maintenance of a healthy gut microbiota [64, 130, 132, 169]. High microbial diversity within the gut microbiota is essential for maintaining metabolic versatility, immune modulation, disease prevention, and overall ecosystem services, which collectively support a healthy gut and contribute to the host’s well-being [8, 66, 90]. Antibiotics are antimicrobial substances that inhibit the growth of or kill microorganisms by targeting pathogenic microbes; however, prolonged use can also cause dysbiosis by depleting beneficial gut microbes, leading to collateral damage when prescribed to treat infections [32, 62, 170]. However, antibiotic resistance in the human gut microbiome is a growing concern, with studies showing the emergence and spread of resistance genes among gut microbiota, impacting both human and animal health [7, 81]. Antibiotic resistance in the intestinal microbiota spreads through mechanisms such as antibiotic-induced gut dysbiosis, which facilitates colonization by resistant bacteria and plasmid-mediated gene transfer, as demonstrated by Yilmaz et al. [173]. Additionally, studies by Loftie-Eaton et al. [89] and [161, 163] show that species-specific plasmid transfer and exposure to nonantibiotic drugs can further enhance the dissemination of resistance genes. These findings underscore the complexity of resistance spread and highlight the need to understand microbiota interactions to address the public health challenge of antimicrobial resistance. Moreover, this need is further emphasized by the World Health Organization’s updated Bacterial Priority Pathogens List (BPPL) for 2024. This list categorizes 15 families of antibiotic-resistant bacteria into critical, high, and medium priority groups to guide research and development for new antibiotics and address antimicrobial resistance. Notably, BBPL reflects changes since the 2017 version and emphasizes the dynamic nature of the issue [96]. The consequences of antibiotic resistance in the gut microbiome include a disruption of the microbial diversity, leading to a decrease in beneficial commensal organisms and an increase in potential pathogens, which can compromise colonization resistance and create opportunities for pathogen invasion into various organ systems [7, 81, 82]. This perturbation can result in dysbiosis, increased antibiotic resistance gene burden, and the potential spread of antibiotic resistance throughout the gut microbiota by horizontal gene transfer, impacting overall health and potentially contributing to the global challenge of antimicrobial resistance [7, 81, 82, 97].

Probiotics have gained significant attention in recent years for their potential health benefits. The World Health Organization (WHO) and the Food and Agriculture Organization of the United Nations (FAO) issued the official definition of probiotics in 2001: “live microorganisms which when administered in adequate amounts confer a health benefit on the host” [46]. ‘Probiotics’ is a useful and accepted term by many organizations, industries, and groups of scientists. The FAO/WHO definition has gained widespread acceptance and shown to be valuable to consumers, regulators, and researchers alike. The FAO/WHO definition of probiotics is used by organizations and agencies like the Institute of Food Technologists [114], Health Canada [19], the World Gastroenterology Organization [59], Codex (which is a division of the FAO/WHO), and the European Food Safety Authority (EFSA).

The International Scientific Association for Probiotics and Prebiotics (ISAPP) panel observed in October 2013 that the probiotic definition “live microorganisms that, when administered in adequate amounts, confer a health benefit on the host” constitutes a more grammatically correct definition [64]. The panel supports the use of this wording going forward. This definition encompasses a wide variety of microorganisms and applications while capturing the core concept of probiotics (microbial, viable and favourable for overall well-being). This definition (with or without the grammatical corrections) has been widely accepted by the scientific community and provides a framework for understanding the role of probiotics in human health.

The worldwide probiotics market was worth USD 46.55 billion in 2020 [112], USD 58.17 billion in 2021 and is predicted to increase at a compound annual growth rate (CAGR) of 7.5% between 2021 and 2030. The market size is projected to reach USD 85.4 billion by 2027 and USD 105.7 billion by 2029 [96]. Food industries, nutritional supplement companies, and specialist probiotic manufacturing companies dominate the market and being pushed by the rising consumer preference for preventative healthcare, as well as the discovery of effective probiotic strains [112]. Furthermore, probiotics are extensively used as culinary additives in dairy diets or supplements in dairy foods such as cheese, yogurt, and cream [22, 28, 95].

## Effects of Probiotics on Gut Microbiota and Health Benefits

**Fig. 1:**
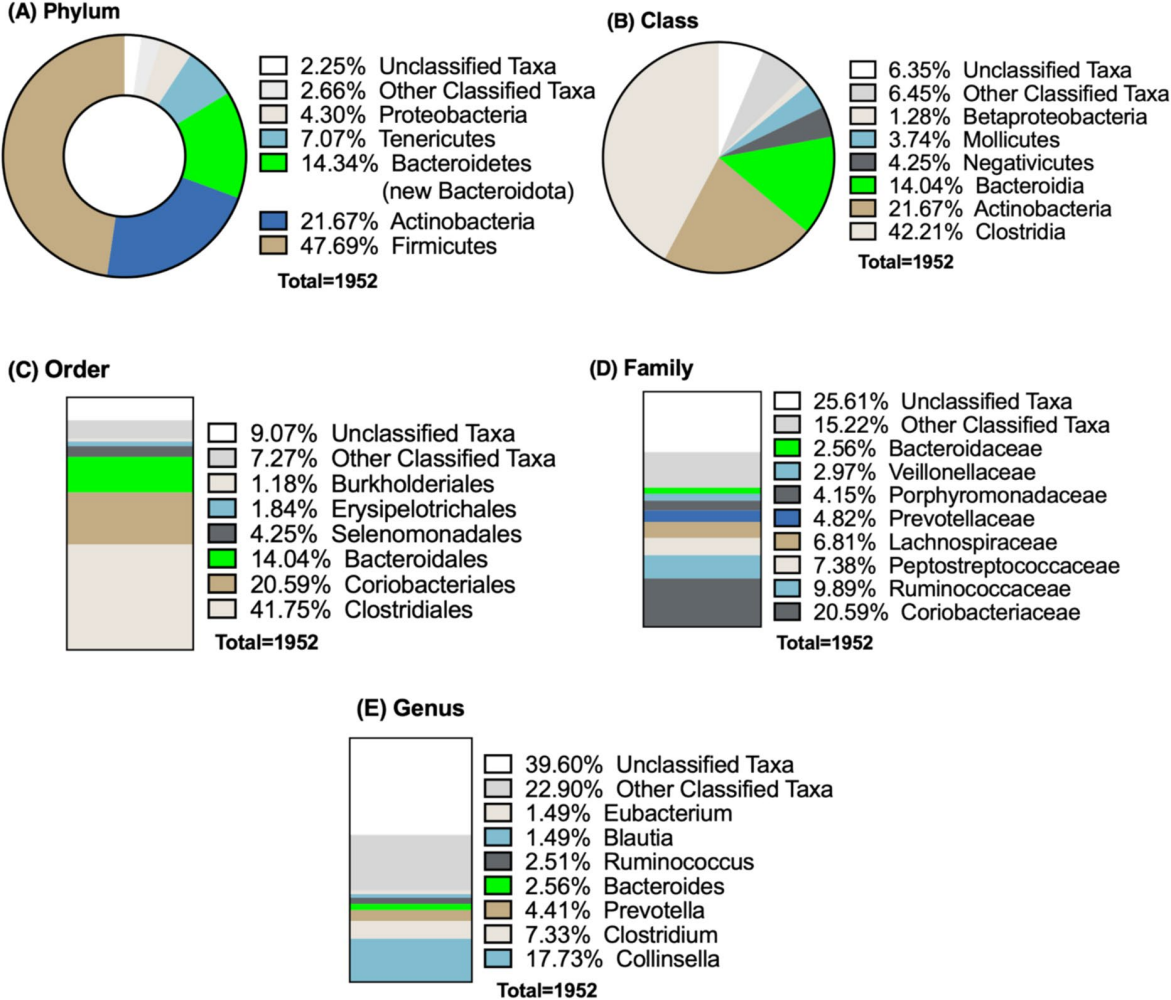
Taxonomy of most abundant non-cultivated bacteria reported by Almeida et al. [5] (A) Bacteroidetes (new Bacteroidota), (B) Bacteroidia, (C) Bacteroidales, (D) Bacteroidaceae, and (E) Bacteroides are highlighted in green color. This figure is showing a total of 1952 uncultured candidate bacterial species by reconstructing 92,143 metagenome-assembled genomes from 11,850 human gut microbiomes data sets from 75 different studies [5]

The human gut contains more than 1000 different types of bacteria, of which more than 50 genera have been identified [1]. The wealth of information now available on the composition of human microbiomes and possible correlates of disease in the age of high-throughput sequencing exceeds any previous understanding of microbial diversity in general, as well as the influence of geography and populations on composition. For example, detailed metagenomic analyses by Almeida et al. [5] recently classified almost 40,000 new MAGs from the previously available global data sets on human microbiomes, leading to the identification of an additional 1952 previously uncultured species for the human microbiome, in addition to the 553 cultured species. This led to a substantial increase in phylogenetic diversity by 281%, the proportion of new *Bacteroidetes* from the previously uncultivated species is over 10% (Fig. 1). With around 10^11^–10^12^ cells per gram, the large intestine has the largest concentration of bacteria in the gastrointestinal system [51]. The establishment and development of the early gut microbiota are believed to be driven by specific compounds present in human milk [103]. Certain genomes of infant gut commensals, particularly bifidobacterial species, are genetically adapted to utilize specific glycans in human milk. This coevolutionary relationship between the host and microbes is thought to benefit both partners [103]. Several studies have linked features of the gut microbiota composition/development during infancy to later health conditions such as asthma, inflammatory bowel disease (IBD), metabolic disorders, and neurodevelopmental disorders [58, 77, 103]. Studies have shown that microbial dysbiosis, characterized by reduced diversity and imbalances in microbial populations, is associated with asthma development. This dysbiosis can disrupt the crosstalk between the gut and airway microbiomes, leading to immune dysregulation and increased inflammation [85, 171]. Specific microbial taxa have been linked to asthma risk. For instance, beneficial bacteria such as certain strains of *Bifidobacterium* and *Lactobacillus* have been shown to modulate immune responses and reduce allergic symptoms in animal models and human studies [79]. Studies indicate that these probiotics can demonstrate anti-inflammatory activity, attenuate airway hyperresponsiveness (AHR), and reduce airway mucus secretion. *Lactobacillus* species, including *L. reuteri* and *L. bulgaricus*, have been specifically noted for their ability to reduce inflammation and modulate immune responses in asthma models [79]. These bacteria can influence the differentiation of regulatory T cells (Tregs), which play a role in maintaining immune tolerance and preventing excessive inflammatory responses [74]. The presence of these beneficial microbes in early life may help establish a balanced immune response, reducing the risk of asthma. The gut microbiota is essential for the development and maturation of the immune system, and early-life events such as maternal influences and antibiotic treatments can significantly impact its composition [60]. These changes may leave a lasting impact, increasing susceptibility to IBD. Specific bacteria and metabolites, such as short-chain fatty acids like butyrate, are vital for gut homeostasis and anti-inflammatory responses. Imbalances can trigger inflammation, leading to IBD [53]. Understanding the relationship between early microbiota and diseases like asthma and IBD is essential for developing preventive strategies, with studies highlighting the need for a diverse, balanced microbiota to support immune health [39].

Reduced diversity or aberrant composition of the infant gut microbiota has been associated with increased risk factors for the above-mentioned adult health conditions [58, 77]. Manipulating the infant gut microbiota through functional food products, such as probiotics, has gained attention as a potential strategy to shape its composition towards a balanced/healthier state. Probiotics can modulate the intestinal microbiota leading to potential control of multiple bowel diseases and promotion of overall wellness [58]. For instance, Jeffery et al. [69] have reported an increase in Firmicutes, particularly *Clostridium*, *Ruminococcus*, and *Dorea*, and a reduction in *Ruminococcus albus*, *Bacteroides fragilis*, *B. vulgates*, and *R. callidus* in irritable bowel syndrome patients. The role of probiotics in shaping the gut microbiome has been investigated extensively. Clinical trials and animal experiments have shown that probiotics can induce changes in microbial composition leading to prevention against pathogen adhesion, improved cognitive function, mood regulation, mental flexibility, stress reduction, modulation of immune responses, improvement in intestinal barrier function, digestion and metabolism enhancement [76, 77, 91, 131, 168].

One area where probiotics have been extensively studied is in the prevention and treatment of gastrointestinal disorders. In particular, there is evidence to suggest that probiotics can be effective in preventing *Clostridium difficile*-associated diarrhoea (CDAD) in adults and children [57]. A systematic review and meta-analysis of randomized controlled trials found that probiotics reduced the risk of CDAD by 60% compared to placebo or no treatment control groups [57]. This finding was supported by another systematic study which also found a reduction in CDAD incidence with probiotic use [29]. In addition to CDAD, probiotics have also been investigated for their potential role in treating acute infectious diarrhoea. Another systematic review including studies on infants showed that while there was some evidence suggesting a reduction in crying time with probiotic use compared to placebo [13]. Probiotics interact with immune cells and intestinal pathogens through a variety of mechanisms that enhance immune responses and maintain gut health. These beneficial microorganisms modulate immune functions by stimulating intestinal immune cells and influencing the balance between immune tolerance and immunogenicity. They achieve this by interacting with host cells in the gastrointestinal tract, including immune, nerve, and endocrine cells, which can lead to enhanced production of short-chain fatty acids and improved gut microbiota balance [65, 101, 181]. Probiotics stimulate immune cells by activating pathways that modulate cytokine production and enhance IgA secretion, improving immune responses [101]. Traditional probiotics, which includes strains or species generally within *Bifidobacterium* or *Lactobacillus* genera, and a few strains from *Bacillus*, *Enterococcus*, *Weissella*, *Saccharomyces*, and *Escherichia coli*, serve important functions in improving host’s health by regulating immune responses [23]. For example, *Bifidobacterium lactis* HN019 improves the immune system of the elderly by activating CD4^+^ and CD25^+^ T lymphocytes and increasing the tumoricidal activities of natural killer cells as well as the phagocytic capacity of mononuclear and polymorphonuclear phagocytes [61]. Another study found *Lactobacillus casei* (Shirota) to be a promising probiotic agent for treating mood and cognitive deficiencies associated with constipation [33, 179]. Also, Bordoni et al. [14] found that the strains *B. bifidum* MB 107 and *B. bifidum* MB 109 are effective for reducing cholesterol levels and Fuglsang et al. [50] found that *Lactobacillus helveticus* CHCC637 and CHCC641 for controlling blood pressure and reduced the heart attack rate in rats. With the recent and significant advancements in bacterial culture methodology and sequencing tools, strains, or species with therapeutic benefits but beyond the spectrum of traditional probiotics have gradually been found and are called next-generation probiotics products. A relatively new bacterial specie, *Akkermansia muciniphila*, showed a negative correlation to diabetes and has the capability to be a promising therapeutic target for obesity. It has shown the potential to increase glucose tolerance and hepatic insulin sensitivity, thereby preventing metabolic endotoxemia in the host [70, 116]. *Faecalibacterium prausnitzii,* one of the major butyrate-producing bacteria in the mammalian intestine, has been shown to control inflammation by inhibiting the nuclear factor kappa B (NF-κB) pathway and generating regulatory T cells, and thus triggers apoptosis in colon cancer cells [108, 116]. These two species (*Akkermansia muciniphila* and *Faecalibacterium prausnitzii*), however, are difficult to cultivate and have yet to be authorized as dietary supplements.

*Bacteroides* is another intriguing contender that has piqued scientists’ interest as a model organism or community for studies of the intestinal microbiota sector and has been considered as the next-generation probiotics [160], owing to their great adaptation features in the host. There are several recent review studies focusing on the probiotic’s characteristics of *Bacteroides* (Table 1). Among the review articles listed in the table below, De Filippis et al. [35] provided a comprehensive overview of the latest advancements in the field of next-generation probiotics. However, it is worth noting that the section dedicated to *Bacteroides* spp. was relatively small and provided limited information. Sun et al. [143] explores the potential of *Bacteroides* as next generation probiotics. However, it is important to note that the review was published in 2019 and may not reflect the latest advancements in the field from the January 2019 to 21.02.2024, which encompass around 1795 according to Scopus databases accessed on February 21st, 2024 with following query *TITLE-ABS-KEY* (“bacteroides” AND “probio*”) *AND PUBYEAR* > *2018* [134]. Since then, there have been significant developments in probiotic research. A comprehensive scan of the primary literature spanning from 2000 to February 21st, 2024, using the search term ‘probiotic*’ yielded an impressive 49,320 articles on Scopus database, with following query TITLE-ABS-KEY (probiotic*) AND PUBYEAR > *1999 AND PUBYEAR* < *2025 AND (LIMIT-TO (LANGUAGE, "English")) AND (LIMIT-TO(DOCTYPE, "ar"))*. In contrast, O’Toole et al. [113] identified 16,064 articles spanning from 2000 to 2016 using the same search term. This indicates a substantial growth in research output during 2016–2024. Consequently, this review aims to provide very recent advancements in the field of probiotics, with a focus on *Bacteroides* spp. until February 2024.

**Table 1:** Recent review papers on probiotic characteristics of *Bacteroides *spp

Study title	Focus of the review	Reference
Next-generation probiotics: the spectrum from probiotics to live biotherapeutics	Covers traditional probiotics, novel microbial therapeutics, regulatory requirements, and future challenges	O'Toole et al. [112]
Investigations of *Bacteroides* spp. towards next-generation probiotics	Explores the potential of *Bacteroides* spp. as next-generation probiotics for immune modulation and inflammation	Tan et al. [149]
A potential species of next-generation probiotics? The dark and light sides of *Bacteroides fragilis* in health	Covers health benefits and risks of *Bacteroides fragilis*	Sun et al. [143]
Safety aspects of next generation probiotics	Highlights the importance of gut microbiota for health and assesses safety of new probiotics concerning antibiotic resistance	Saarela [129]
Roles of intestinal *bacteroides* in human health and diseases	Emphasizes the role of dietary nutrition on the intestinal microbiome and debates on *Bacteroides* spp. roles	Wang et al. [160]
Outlook on next‑generation probiotics from the human gut	Highlights the need for safety studies, personalized applications, and challenges in cultivation and storage	De Filippis et al. [35]

## Why *Bacteroides* spp. are Potential Candidates for Probiotics?

**Fig. 2:**
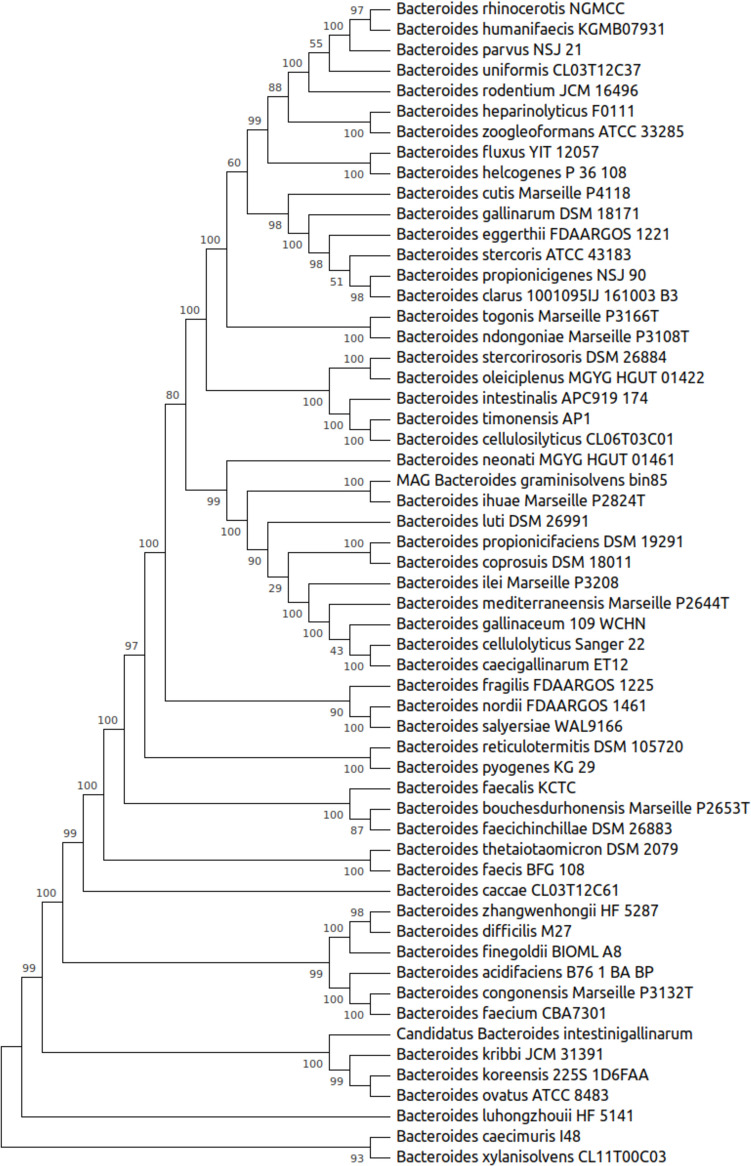
Phylogenetic tree of evolutionay relationship of the representative species (57) among *Bacteroides* genus. Whole genome sequences of the representative/reference genomes were downloaded from NCBI database [111] using NCBI command line tools. The phylogenetic tree was constructed using phylogenomics workflow of Anvi’o v8 [43], which included Bacteria_71 collection of single-copy core genes as HMM profile, which also contains ribosomal proteins. The phylogenetic tree was further optimized using MEGA v11.0.13 [148]

Bacteroidota (formerly known as Bacteroidetes) is a major phylum within the gut microbiota, accounting for a significant proportion of the intestinal microbial community in healthy adults. This phylum includes genera such as *Bacteroides*, *Parabacteroides*, *Prevotella*, and *Alistipes*, which are crucial for maintaining intestinal homeostasis and overall human health. Studies have shown that Bacteroidota can constitute between 20 and 80% of the gut microbiota, emphasizing its importance in the gastrointestinal ecosystem [151]. Bacteroidota plays a vital role in the digestion of complex carbohydrates, leading to the production of short-chain fatty acids (SCFAs) such as butyrate. SCFAs are essential for colonocyte energy, maintaining the gut barrier, and exerting anti-inflammatory effects. This phylum is involved in modulating the host immune system by influencing the maturation of immune cells and cytokine production. Such modulation is crucial for immune tolerance and protection against pathogens [93]. Imbalances in Bacteroidota have been linked to various health conditions, including inflammatory bowel disease (IBD) and metabolic disorders. Their role in maintaining gut homeostasis makes them a focus for potential therapeutic interventions [10, 93]. A diverse Bacteroidota population contributes to a balanced gut microbiome, preventing the overgrowth of pathogenic bacteria and maintaining a stable microbial ecosystem [52]. Bacteroidota engages in complex metabolic interactions with other gut microbes, facilitating the breakdown of dietary fibers and the production of beneficial metabolites. This cooperation supports nutrient absorption and gut health [10]. The composition of Bacteroidota can be influenced by dietary factors, affecting their functional roles in the gut. Certain dietary fibers can promote the growth of specific Bacteroidota species, enhancing their beneficial effects [10],Y. [83, 84]. King et al. [78] reported that Bacteroidota is a major phylum with 18.9% members in the healthy human gut based on 48 fecal samples from 16 healthy volunteers at the George Washington University Foggy Bottom campus area, Washington, D.C., United States. The most prevalent gram-negative bacterial order, *Bacteroidales*, has 29 species and may be found in the human colon at high concentrations of 10^9^–10^11^ CFU per gram of faeces. Members of this order demonstrate closer genetic relationship to each other (Fig. 2), compared to those in other bacterial orders [107]. Additionally, *Bacteroides* is one of the main genera of the core microbiota category, and among its species, *Bacteroides uniformis*, *Bacteroides caccae*, *Bacteroides vulgatus*, and *Bacteroides thetaiotaomicron* have relative abundances of above 1% in the human gut [67, 71, 72]. However, the *Bacteroides* genus exhibits substantial variation in diversities across individuals [20, 75, 123].

*Bacteroides* is a Gram-negative and obligate anaerobic bacterial genus. *Bacteroides* are non-endospore forming bacilli that can be motile or nonmotile depending on the species [129]. Their Gram-negative and anaerobic nature significantly influences their interactions with the host and their potential as probiotics. Bacteroides thrive in the oxygen-deprived environment of the gastrointestinal tract. This anaerobic nature allows them to efficiently break down complex carbohydrates and contribute to the host’s energy supply such as SCFAs (butyrate), which are beneficial for colon health and have anti-inflammatory properties [117]. The outer membrane of Gram-negative bacteria like Bacteroides contains lipopolysaccharides (LPS), which can interact with the host’s immune system. While LPS is generally considered a pathogen-associated molecular pattern (PAMP) that can trigger immune responses, Bacteroides species have evolved mechanisms to modulate these responses, often promoting immune tolerance and maintaining intestinal homeostasis [21, 136]. Bacteroides species are generally mutualistic, constituting most of the mammalian gastrointestinal microbiota and playing an important role in the host intestine by conversion of complex molecules (organic polymers) to simpler ones, and by providing nutrition and vitamins [92, 167, 175]. Moreover, *Bacteroides* spp. play a crucial role in detoxifying bile acid chains, returning the acid to the liver circulation. They also have an impact on the regulation of T cells during inflammation by producing a polysaccharide called polysaccharide A (PSA, have important immunomodulatory effects) [17].

### Polysaccharide Degradation by *Bacteroides* spp. and Gut Health

The ability of *Bacteroides* species to degrade polysaccharides is well known. *Bacteroides* produce short-chain fatty acids (SCFAs) such as butyrate, propionate, acetate, and formate by degrading non-digestible animal or plant-based glycans obtained from daily food intake or intestinal mucus [172]. These SCFAs offer numerous health benefits, such as reducing inflammation and preventing cancer [122]. Besides, *Bacteroides* possess carbohydrate-active enzymes (CAZymes) that break down complex polysaccharides in dietary fiber, such as cellulose, hemicellulose, and pectin. These enzymes convert polysaccharides into smaller sugar units like glucose, fructose, and xylose. *Bacteroides* metabolize these sugars through various pathways, with glycolysis being key in producing SCFAs such as acetate, propionate, and butyrate. *Bacteroides* spp. produce precursors utilized by other butyrate producers, enhancing overall butyrate levels in the gut. They break down dietary polysaccharides into simpler compounds that can be fermented into butyrate. Some Bacteroides strains have genes encoding enzymes involved in direct or indirect butyrate production [48, 126, 157]. SCFAs serve as essential energy sources for gut epithelial cells, support intestinal barrier function, regulate immune responses, and promote gut health [30],S. [161, 163].

*Bacteroides thetaiotaomicron* is an important member of the human gut microbiota that helps break down dietary polysaccharides and produce SCFAs [26]*. B. thetaiotaomicron* contains two-component system proteins that aid in the induction of the expression of enzymes involved in polysaccharide degradation in response to environmental signals sensed by the sensing protein [45], and the switch of gene subsets during utilization of various substrates is determined by their availability and preference [73]. Additionally, *Bacteroides* spp. including* B. ovatus* [26]*, B. fragilis* [166], *B. uniformis*, and *B. vulgatus* are few examples that possess CAZymes and are known to degrade polysaccharides and produce SCFAs [26].

*Bacteroides* species’ polysaccharide consumption mechanisms also boost their ecological viability by ensuring a diverse range of target polysaccharides, particularly non-digested food fibers. According to a study, the simple glycoprotein produced by xylan could aid in the growth of probiotics such as *Bifidobacterium* [9]. Most *Bacteroides* spp. will not digest mucin unless it is the sole nutrition source available for development, which mainly excludes the pathogenic potential of *Bacteroides* spp. to directly target the host’s protective intestinal barrier [106]. *Bacteroides* polysaccharide utilization gene cluster are made up of homologs of the starch-utilization system (Sus), such as SusC and SusD, which bind the target glycans and induce over 200 genes encoding glycoside hydrolases, polysaccharide lyases or transferases, which depolymerize the target glycans [34, 44].

### Anti-Inflammatory Role of *Bacteroides* spp.

*Bacteroides* spp. have demonstrated the ability to modulate the function of immune cells, specifically dendritic cells, and macrophages, thereby promoting an anti-inflammatory phenotype [64]. Through their influence on the activation and polarization of these immune cells, *Bacteroides* play a crucial role in mitigating inflammation, which holds significant implications for gut health and the prevention of gastrointestinal disorders [175].

*Bacteroides fragilis* is a prevalent species within the human gut microbiota. This Gram-negative anaerobic bacterium is renowned for its pathogenic capabilities in specific circumstances. However, certain strains of *B. fragilis* have been linked to advantageous effects, including immune regulation and anti-inflammatory properties [6]. Polysaccharide A (PSA) derived from *B. fragilis* is a capsular carbohydrate that exhibits significant T cell-dependent pro- and anti-inflammatory properties. Recent studies have demonstrated the ability of PSA to effectively suppress inflammatory bowel disease and experimental autoimmune encephalomyelitis through the MHCII-dependent activation of CD4 + T cells [6, 109]. Furthermore, in another study, anti-inflammatory and epithelium reinforcing *Bacteroides* and *Parabacteroides* spp. were isolated from a healthy faecal donor [64]. These findings indicated that *Bacteroides ovatus* exhibited effectiveness in reducing inflammation and mitigating colitis [64]. Additionally, *Bacteroides thetaiotaomicron* possesses notable anti-inflammatory properties and has demonstrated the ability to enhance mucosal barrier function, restrict pathogen infiltration, and alleviate colon inflammation in preclinical models of Crohn’s disease [37]. Overall, *Bacteroides* spp. play a crucial role in maintaining gut health through their diverse anti-inflammatory effects. These effects include modulating immune cell function, possessing anti-inflammatory capsular polysaccharides, and promoting beneficial effects on the immune system through *Bacteroides fragilis* polysaccharides. These anti-inflammatory properties are essential for preventing gut disorders and supporting overall gut health [64, 159, 175].

### Protection from Pathogens

*Bacteroides* spp. constitute gut commensals with the incredible ability to offer protection against pathogens. Their presence in the gut ecosystem contributes to protecting it from colonizing of detrimental bacteria that may cause infections or various diseases. As important gut commensals, *Bacteroides* spp. fulfil various key functions, such as providing nutrients to fellow microbial inhabitants, actively compete and occupy limited niches, limiting resources for potential pathogens and ensuring the overall balance of the gut microbiota [175]. This competition prevents growth of harmful bacteria, protecting the host from invasion.

*Bacteroides* spp. possess the ability to produce antimicrobial substances that effectively impede the growth or directly kill specific pathogens. One significant example is the secretion of bacteriocins, which are small antimicrobial peptides designed to selectively target bacterial species [175]. For example, *B. fragilis* produces fragilysin, a bacteriocin that effectively inhibits the growth of harmful bacteria within the gut [175]. Through the production of these substances, *Bacteroides* spp. play a pivotal role in upholding the balance of the gut microbiota and preventing the excessive proliferation of harmful pathogens.

### Anti-Carcinogenic Role of *Bacteroides* spp.

Research on the anti-cancer impact of *Bacteroides* spp. is rapidly advancing, and although the precise mechanisms are not yet fully understood, compelling evidence strongly indicates significant anti-cancer effects correlated with these bacteria. *Bacteroides* spp., along with other gut bacteria, contribute to the production of butyrate and other SCFAs. These SCFAs, especially butyrate, exhibit potent anti-carcinogenic effects, particularly in the colon [161, 163]. Butyrate supplies as an energy source for colonocytes and plays a vital role in preserving the integrity of colonic epithelium. This function is crucial for gut health and the prevention of gastrointestinal disorders, including colorectal cancer [105, 161, 163]. Overall, by promoting a healthy gut environment, *Bacteroides* spp. indirectly support butyrate production, which may help prevent colorectal cancer development.

Furthermore, Dysbiosis, an imbalance in the gut microbiota, has been consistently associated with an increased risk of cancer, particularly colorectal cancer [135]. *Bacteroides* spp. are thought to play a role in maintaining a diverse and balanced gut microbiota, which is believed to have a protective effect against certain types of cancer, including colorectal cancer [125]. An investigation was conducted to explore the association between *B. thetaiotaomicron* and colorectal cancer. The findings indicated a potential protective effect of *B. thetaiotaomicron* against colorectal cancer through the promotion of a healthy gut environment and the reduction of inflammation. These results suggest the involvement of *B. thetaiotaomicron* in mitigating the risk of colorectal cancer by fostering a favourable gut milieu and suppressing inflammatory processes [150]. Despite the recent advances in the subject area, the relationship between *Bacteroides* spp. and cancer is complex, requiring further research to fully understand their specific roles. Effects may vary depending on species, strains, host factors, and gut microbiota interactions. More studies are needed to uncover the mechanisms behind *Bacteroides* spp.’s potential anti-carcinogenic properties and explore their applications in cancer prevention and treatment.

## Genetic Engineering to Improve Probiotic Potential

Genetic engineering has opened up new avenues for improving the potential of probiotics for human health [87]. The necessity of employing genetic engineering in the design of probiotics stems from the urgent need to enhance the efficacy and spectrum of benefits these microorganisms can offer to human health. Probiotic strains selected through conventional methodologies may not consistently exhibit the requisite characteristics necessary for beneficial interactions within the complex human microbiome; consequently, current probiotics demonstrate several limitations. The survival of probiotics through the gastrointestinal tract and their ability to colonize the gut effectively is a significant challenge due to the environmental factors and the host's existing microbiota which can impact their viability and efficacy [144, 145]. The effects of probiotics are often strain-specific, making it difficult to generalize findings across different strains or species, which necessitates detailed research to identify the most effective strains for specific health outcomes [137]. In addition, there are regulatory hurdles and safety concerns related to the use of live microorganisms, particularly in immunocompromised individuals or those with underlying health conditions [144, 145]. Genetic engineering offers promising solutions to overcome these limitations by enhancing the functionality and stability of probiotics. Engineered probiotics can be designed with specific traits, such as biosensing abilities, enhanced stability and viability, and tailored health benefits [38, 68, 87, 128]. The incorporation of genetic engineering allows for the precise manipulation of probiotic strains, enabling the introduction of new characteristics or the enhancement of existing ones. Probiotics can be engineered to produce and deliver therapeutic proteins, peptides, or small molecules directly to specific sites in the body [18, 36, 80]. This targeted delivery increases the efficacy and reduces potential side effects compared to conventional drug administration. In addition, probiotics can be engineered to possess enhanced immunogenic characteristics, enabling them to modulate the immune system more effectively for therapeutic purposes like treating autoimmune diseases or boosting immunity [36, 80, 99]. Genetic circuits and biosensors can be introduced into probiotics, allowing them to detect and report the presence of specific disease biomarkers or conditions with high precision [36]. This could lead to less invasive and more cost-effective diagnostic methods. Next-generation probiotics, enhanced through genetic engineering, offer promising solutions to address challenges such as antibiotic resistance and colonization capacity in the gastrointestinal tract. By utilizing CRISPR-Cas systems, these advanced probiotics can be engineered to carry plasmid vaccines that prevent the spread of antibiotic resistance genes among gut microbiota. This approach not only reduces the prevalence of resistant strains but also enhances the overall health of the microbiome [3, 98]. Additionally, genetic modifications can improve the colonization capacity of probiotics by enabling them to respond dynamically to specific environmental cues within the gut [2, 41]. Furthermore, advanced coating strategies, such as nanoarmor, have been used to enhance the stability and mucoadhesive capacity of probiotics, ensuring they remain viable and can effectively target inflamed sites within the gastrointestinal tract [183].

Probiotics can also be engineered to produce and deliver anticancer compounds or to selectively target and kill cancer cells, offering a potential new avenue for cancer treatment [99]. Genetic engineering can be used to remove antimicrobial resistance genes or introduce other modifications to enhance the safety and efficacy of probiotic strains for human consumption [99]. Moreover, benefits of genetic engineering also include improved survival rates of probiotics in the harsh conditions of the gastrointestinal tract, increased production of beneficial metabolites like vitamins or short-chain fatty acids, and the specific targeting of pathogenic bacteria while sparing beneficial microbes, ultimately leading to probiotics that are more effective and tailored for specific health outcomes [36, 80, 99]. One of the pivotal techniques in genetic engineering is gene editing via the CRISPR-Cas system. This revolutionary methodology has transformed the landscape of molecular biology and biotechnology, by providing an unprecedented level of precision, efficiency, and versatility in editing the genetic code of organisms across the domains of life. The presence of CRISPR-Cas systems is observed in 85.2% of archaeal species and 42.3% of bacterial species [121]. The occurrence of this CRISPR-Cas system significantly differs across various probiotics, for example, more than 40% of *Lactobacillus* genus harbor complete CRISPR-Cas systems [127], and 57% of *Bifidobacterium* strains possess CRISPR-Cas systems, encompassing five distinct subtypes: Type I-E, Type I-C, Type I-G, Type II-A, and Type II-C [115]. In another study, Tajkarimi and Wexler [147] found that among the *Bacteroides fragilis* (*n* = 109) strains analyzed, 97% exhibited one or more CRISPR-Cas systems, grouped into four main types: Class 1 Type IB, Class 1 Type IIIB, Class 2 Type IIC, and an additional array without associated cas genes. CRISPR-Cas technology has significantly advanced probiotic development by enabling precise genome editing in strains like *Lactobacillus* and *Bifidobacteria*, leading to enhanced therapeutic potentials and functionalities for improved human health [155]. CRISPR-Cas based genetic engineering improved cell growth and lactic acid production in *Pediococcus* *acidilactici* [88]. The engineered CRISPR-Cas9 system developed in *Lactobacillus acidophilus* NCFM, *Lactobacillus gasseri* ATCC 33323, and *Lactobacillus paracasei* Lpc-37 strains can facilitate targeted gene deletion, insertion of specific sequences such as the mCherry fluorescent-protein gene, and create single-base substitutions. This system enables precise genome editing with high mutant recovery rates, demonstrating its versatility and efficiency for programmable genome engineering in these *Lactobacillus* species and also phylogenetically distant *Lactobacillus* species [56]. In addition, *Bacillus subtilis*, generally regarded as safe (GRAS) [156], has been subjected to extensive genetic modifications through CRISPR-Cas9 technologies starting from 2016 [138, 164, 184]. An important report by Zheng et al. [180] introduces a ground-breaking CRISPR/Cas-based genome editing tool specifically designed for *Bacteroides* species. By employing the highly efficient FnCas12a system, the tool enables precise, markerless gene editing, including large genomic deletions and insertions across multiple *Bacteroides* species. This advancement significantly propels the development and functional investigation of *Bacteroides*-based probiotics, marking a pivotal leap forward in gut microbiome research. Deactivated Cas9 (dCas9)-based CRISPR interference has been effectively utilized for targeted knockdown in *Bacteroides* *thetaiotaomicron* [104]. Drawing from prior research, we suggest that the CRISPR/Cas system is capable of performing genome editing in *Bacteroides* species. Through genetic engineering, the potential of probiotics can be significantly expanded, paving the way for novel therapeutic applications and a deeper understanding of their role in human health. Genetically engineered probiotics, despite their potential health benefits, raise concerns including safety, regulatory hurdles, ecological impact, and public acceptance; therefore, they are not currently allowed to be used in humans [102, 141]. Addressing these requires rigorous testing, stringent regulatory adherence, and transparent dialogue among scientists, regulators, and the public to ensure their safe and beneficial use [102].

## Clinical Evidence Supporting the Use of *Bacteroides* spp. as Probiotics

Extensive research has been conducted on *Bacteroides* spp., highlighting their significance. However, it is important to note that the use of *Bacteroides* spp. as probiotics is an emerging field but still in its infancy. Most studies have focused on their natural role in the gut microbiota rather than their direct use as probiotics. Nevertheless, some in vitro and in vivo (animal) studies provide insights into the potential benefits of *Bacteroides* spp. Other bacterial genera which are considered as probiotics are *Lactobacillus*, *Bifidobacterium*, *Bacillus*, and *Saccharomyces*, each demonstrating various health benefits such as enhancing gut health, modulating immune responses, and preventing gastrointestinal disorders [120]. These probiotics are known for their ability to modulate the immune response, enhance gut barrier function, and compete with pathogens for colonization sites. For instance, *Lactobacillus* and *Bifidobacterium* species are commonly used to improve gastrointestinal health and have been shown to remain stable in various delivery matrices, such as human milk, which can complement the health benefits of breastfeeding [94]. *Saccharomyces* spp., particularly *Saccharomyces cerevisiae*, are used in both human and animal health to improve gut health and combat infections [47].

Clinical evidence shows that *Bacteroides* genus as compared to *Lactobacillus*, *Bifidobacterium*, *Bacillus*, and *Saccharomyces* has significant advantages as probiotics due to their unique ability to thrive in diverse environments and their potential to produce short-chain fatty acids, which play a crucial role in maintaining gut health and reducing inflammation [185]. Recent laboratory studies have revealed that certain strains of *Bacteroides* spp. possess anti-inflammatory and epithelium-strengthening properties [64]. These studies have found that strains belonging to *B. caccae*, *B. intestinalis*, and *B. fragilis* can reduce inflammation and enhance the epithelium. Cultivation tests have also shown that *Bacteroides* spp. are sensitive to metronidazole, carbapenems, and beta-lactam/beta-lactamase inhibitor combinations [118]. However, it’s important to note that some *Bacteroides* strains are now highly resistant to tetracyclines and commonly used beta lactams (penicillins and cephalosporins and derivatives) primarily due to acquiring resistance genes through horizontal gene transfer and high selection pressure [124]. Additionally, lipopolysaccharides derived from certain *Bacteroides* spp. have been found to affect the immune system [165]. Overall, these recent studies indicate that specific strains of *Bacteroides* spp. may have potential therapeutic benefits, especially in the context of gut microbiota research.

Recent animal studies have provided compelling evidence supporting the potential viability of *Bacteroides* spp. as valuable probiotics. A study by Zhou et al. [182] revealed that the administration of *B. fragilis* strain ZY-312 effectively mitigated radiation-induced intestinal injury in mice, as evidenced by reduced weight loss, shortened intestinal length, and improved intestinal permeability. Another study found that a *B. fragilis* demonstrates the capacity to preserve gut integrity and inhibit inflammation in a mouse model [139]. The non-toxic strain of *B. fragilis*, ZY-312, has also been shown to provide protection against antibiotic-associated diarrhoea (AAD) in rats. The study by Zhang et al. [178] reveals that treatment with *B. fragilis* ZY-312 alleviates AAD symptoms by modulating the gut microbiota, enhancing epithelial cell organization, and improving barrier function. These effects result in reduced inflammation and the restoration of gut homeostasis. Collectively, these initial studies already indicate that *Bacteroides* spp. hold promise in promoting intestinal barrier integrity, mitigating inflammation and antibiotic-associated diarrhoea, and modulating the host immune response. Nonetheless, further research is needed to comprehensively understand the impact of *Bacteroides* spp. on the animal host.

## Safety Considerations and Regulation of *Bacteroides* spp. as Probiotics

The safety considerations of using *Bacteroides* spp. as probiotics are an important aspect to explore before their widespread use. While Bacteroides species have shown potential as probiotic candidates, it is crucial to evaluate their safety profile to ensure they do not pose any risks to human or animal health. When considering the safety of probiotics, several factors need to be considered, such as the strain-specific characteristics of *Bacteroides* spp., potential interactions with the host immune system, and the potential for adverse effects. It is also essential to assess their potential for pathogenicity and antibiotic resistance. Although the majority of *Bacteroides* species are harmless, certain strains have the capacity to transform into opportunistic pathogens. Such strains can give rise to infections, particularly in individuals with compromised immune systems [175]. Consequently, if an unsuitable strain or an imbalanced population of *Bacteroides* spp. is introduced as a probiotic, it has the potential to result in infections or other health complications. Owing to the high inter-individual variability in the human gut microbiome, a one-size-fits-all probiotic formulation may not be effective across all individuals. The gut microbiome variation, stemming from factors like diet and genetics, can influence an individual’s response to empiric probiotic interventions. Therefore, next-generation probiotics engineered via synthetic biology hold promise in developing personalized probiotic therapies tailored to target specific pathways based on an individual’s distinct microbiome profile for optimized therapeutic outcomes.

In the context of the intricate gut ecosystem, where a diverse array of microorganisms coexists, it is essential to consider the potential risks posed by introducing excessive quantities of *Bacteroides* spp. through probiotics without accounting for the overall balance of the gut microbiota. This lack of consideration, especially in cases of excessive consumption in the worst case, can lead to a disruption of the delicate balance, resulting in dysbiosis-an imbalance associated with various health conditions, including inflammatory bowel disease, obesity, and metabolic disorders. For example, a study identified seven *Bacteroides* spp., three (*B. cellulosilyticus*, *B. salanitronis*, and *B. dorei*) serve as probiotics, while four (*B. thetaiotaomicron*, *B. vulgatus*, *B. ovatus*, and *B. fragilis*) are pathogenic. The study emphasizes the importance of maintaining a specific bio-geographical location within the gut to ensure these organisms remain in their intended habitat and avoid contact with host tissues [175]. Therefore, prior to their introduction as probiotics, it is crucial to thoroughly assess the safety and effectiveness of each individual strain. According to another study, alterations in the Firmicutes/Bacteroidetes (F/B) ratio can also contribute to obesity or inflammatory bowel disease [142]. Fortunately, specific probiotics have the potential to restore the balance of the gut microbiota. Nevertheless, it is crucial to take caution when introducing substantial amount of *Bacteroides* spp. through probiotics, as disregarding the overall balance of the gut microbiota may disrupt the existing microbial community, resulting in adverse health conditions [142]. Furthermore, safety assessments must include preclinical studies and rigorous clinical trials to evaluate the tolerability and safety of *Bacteroides* spp. as probiotics in different populations, including healthy individuals and those with specific health conditions. These studies should assess parameters such as gastrointestinal tolerability, immune response, and potential allergic reactions.

In contrast to traditional probiotics like *Lactobacilli* and *Bifidobacteria*, which are widely recognized as Generally Regarded as Safe (GRAS) in the USA and approved for consumption by the European Food Safety Authority, the beneficial effects of Bacteroides vary significantly across different strains which can also include toxic strains or toxic effects of beneficial strains [112]. While non-toxigenic strains of *B. fragilis* can mitigate infections and aid in gut colonization, their polysaccharides can also support bacterial growth and have been associated with abscess formation [86, 100, 146]. Furthermore, strains of *B. fragilis* that possess the virulence-associated genes encoding fragilysin can lead to enhanced inflammation and severe forms of colitis [174]; additionally, the *B. fragilis* YCH46 strain, which produces a fibrinogen-degrading protease, has the potential to weaken the body’s defense mechanisms and increase susceptibility to infections through bacterial invasions in injured tissues [25]. In another study, Brook [16] underscored the critical importance of identifying and evaluating the strains of beta-lactamase-producing bacteria in head and neck infections. These studies emphasize the necessity for meticulous strain identification and rigorous safety assessments in both scientific research, clinical and industrial application.

Consequently, the FAO/WHO firmly advocates for a tri-phase clinical trial process prior to the utilization of probiotics. This process encompasses an evaluation of safety and functionality, double-blind, randomized, placebo-controlled studies involving humans, and a comparison of effectiveness with conventional treatments [46]. Nonetheless, advancements in the investigation of microorganisms with potential health benefits indicate a need for updating the guidelines for their industrial use. Therefore, in 2016, the US Food and Drug Administration (FDA) declared that emerging probiotics ought to be initially formulated as dietary ingredients instead of dietary supplements. Furthermore, it stated that live biotherapeutic products, defined as: “a biological product composed of live organisms for prevention, treatment, or cure a disease or condition of human beings but is not a vaccine”, should undergo the investigational new drug process [113, 130, 132].

The European Commission, highlighting the latest advancement under Novel Food Regulation (EC) No 258/97, issued its most recent approval in 2015 for *B. xylanisolvens* DSM 23964, a member of the *Bacteroides* genus, as a fermentation starter for pasteurized milk products. This approval allowed the market introduction of pasteurized milk products fermented with this bacterium and subsequently pasteurized as novel food, provided they are subjected to heat treatment to render the bacteria non-viable [15, 42]. This particular strain has never been used in the food industry before, and no strains belonging to the genus *Bacteroides* are known to have a verified history of application in food manufacturing. Therefore, this is a notable example of regulatory approval which underwent extensive testing for non-pathogenicity through various in vitro and in vivo studies before being authorized for use in food products by the European Commission [15, 42]. The safety of the bacteria was evaluated by examining extracellular enzymes, pathogenic factors, and antibiotic resistance in vitro, alongside hematological parameters, serum inflammatory markers, and liver enzyme levels in both animal and human trials, to confirm its non-pathogenic nature [153, 154], Philippe [152, 153, 154]. This safety assessment was further validated by the German Federal Institute for Occupational Safety and Health (Bundesanstalt für Arbeitsschutz und Arbeitsmedizin, BAuA), confirming the non-pathogenic status of the bacterium [11]. Additionally, *B. xylanisolvens* must be rendered inactive in its final form within actual products and is still pending inclusion on the Qualified Presumption of Safety (QPS) list published by EFSA (accessed on 06.03.2024 https://zenodo.org/records/10534041).

**Fig. 3:**
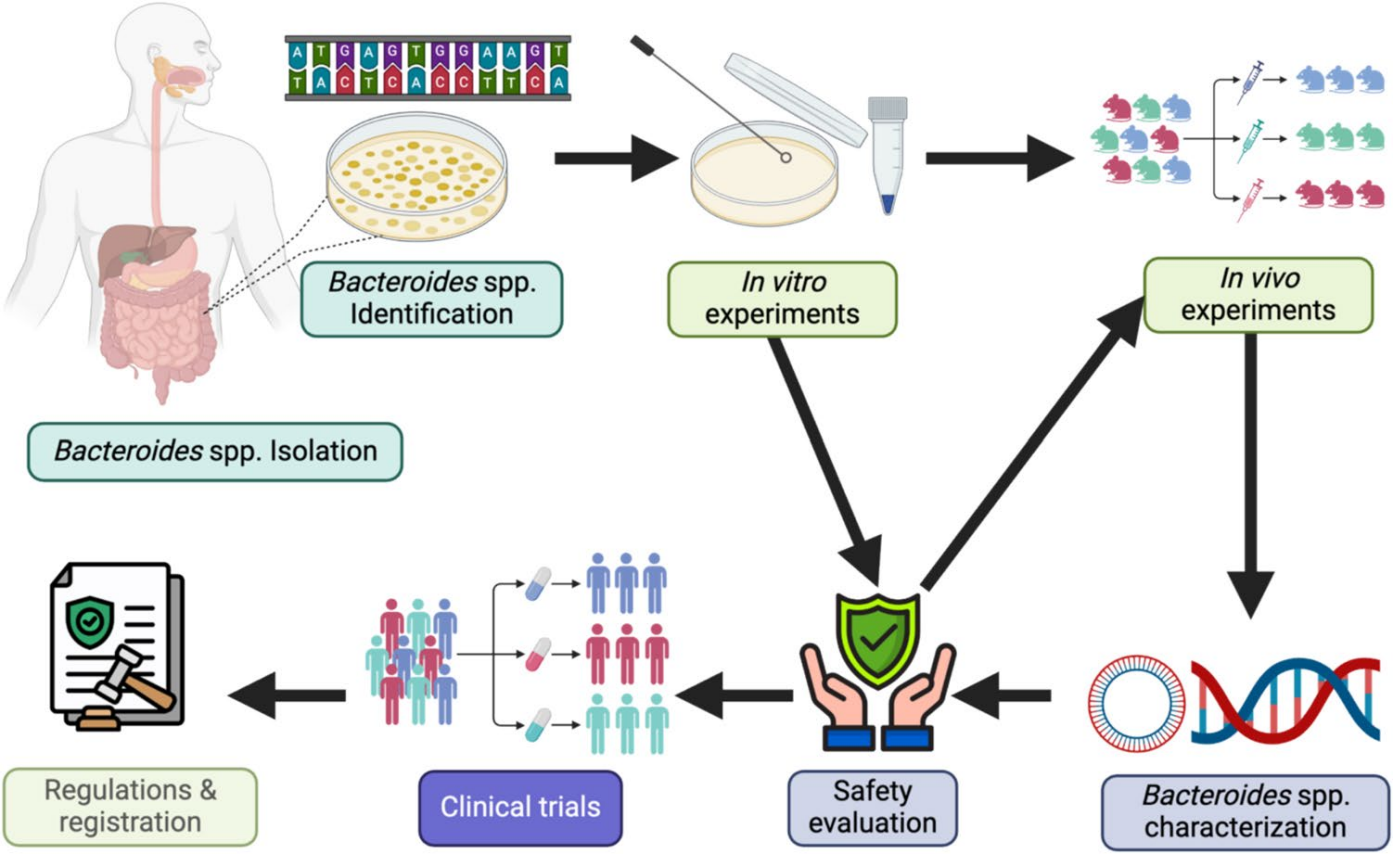
A flowchart diagram illustrating the development process for *Bacteroides* spp. being prepared for use as next-generation probiotics prior to approval for commercial use [112]. Created with BioRender.com. ***Bacteroides* spp. Isolation:** This is the starting point where *Bacteroides* spp. are isolated, from human faecal samples. ***Bacteroides* spp. Identification:** Following isolation, the species undergo identification procedures, which likely involve culturing the bacteria on plates on specific media and molecular identification via 16S rRNA Sequencing. **In vitro Experiments:** Once identified, the bacteria are subject to in vitro experiments, which are tests conducted in a controlled environment outside of a living organism such as acid and bile tolerance, epithelial cell adherence, antimicrobial activity, antibiotic resistance screening, resistance to gastrointestinal enzymes, immunomodulatory effects, competitive exclusion, genetic stability, production of beneficial metabolites, cellular toxicity testing, stress resistance, and functional attribute analysis. **In vivo Experiments:** Subsequent to in vitro testing, in vivo experiments are conducted, which involve testing on living organisms. These tests include T cell regulation, serum metabolite, intestinal integrity, colonization efficacy, pathogen challenge tests, immunomodulation studies, safety assessment, therapeutic efficacy tests, long-term stability and efficacy, dosage optimization, drug and dietary component interactions. ***Bacteroides* spp. Characterization:** The results from both in vitro and in vivo experiments lead to the characterization of the *Bacteroides* strains. This involves analyzing and documenting their genotypic and phenotypic properties. **Safety Evaluation:** This is part of the clinical trials where the safety of the Bacteroides strains is thoroughly assessed to ensure that no adverse effects occur. **Clinical Trials:** Alongside or after characterization, clinical trials are conducted to evaluate the safety and efficacy of the Bacteroides probiotic in humans. This step is crucial before any probiotic can be considered for market release. **Regulations & Registration:** This step involves the legal and regulatory aspects where the probiotics are reviewed against standards and guidelines, presumably by bodies like the FDA, EFSA, and local or international bodies, before they are approved for industrial application. These guidelines were used also for the registration of *B. xylanisolvens* DSM 23964 [15]

The process of evaluation and regulation depicted in Fig. 3 establishes a benchmark for meticulously evaluating and validating Bacteroides strains for prospective health benefits, underscoring the critical need to weigh the advantages against potential hazards in the development of next-generation probiotics.

In recent years, the potential of *Bacteroides* strains as probiotics has garnered significant attention due to their beneficial effects on human health. To provide a comprehensive overview of the current research landscape, we have compiled a detailed summary of studies investigating various *Bacteroides* strains (Table 2). These strains have been explored for their probiotic potential, characterized by their source of isolation, beneficial mechanisms, as well as through various study models and safety evaluations. Table 2 showcases potential Bacteroides strains that have emerged as promising candidates for advanced probiotic development, which have not been mentioned in previous review articles as show in Table 1. Therefore, Table 2 not only highlights the diversity of sources and beneficial effects associated with *Bacteroides* strains but also underscores the rigorous characterization and safety assessments undertaken in these studies.

**Table 2:** Potential *Bacteroides* strains as candidates for advanced probiotic development

*Bacteroides* strain	Source of isolation	Genome sequence	Beneficial mechanism	Characterization	Study model	Safety evaluation	Reference
*B. fragilis* LTBF12	Healthy human from a long-lived population in Guangxi, China	No	Amelioration of anxiety and necrosis of hippocampal neuronal cells in senescent mice, improvement of antioxidant capacity, reduction of inflammation levels	Simulated gastrointestinal tolerance, self-aggregation capacity, and surface hydrophobicity testing	Naturally ageing C57BL/6J mice	-	[83, 84]
*B. dorei* BDX-01	Faeces of a healthy human	No	Alleviates DSS-induced experimental colitis, regulates intestinal bile salt hydrolase activity, increases intestinal bile acid excretion and deconjugation, modulates gut microbiota composition, activates the colonic farnesoid X receptor (FXR), downregulates proinflammatory cytokines, suppresses the NLRP3 inflammasome signaling pathway, and improves symptoms and pathological damage of acute colitis	Sulturing on BHI agar with defibrinated sheep blood under anaerobic conditions, morphophysiological characteristics matching *B. dorei*, identification of 14 virulence factor homologs related to cellular structure or physiological activities, absence of pathogenesis-related virulence factors, no plasmids found in the genome, antibiotic sensitivity to cefoperazone, meropenem, tetracycline, ciprofloxacin, clindamycin, clarithromycin	Caco-2 and IEC-6 cell lines, and a dextran sulfate sodium (DSS)-induced colitis model in C57BL/6 mice	In vitro and in vivo safety, antibiotic resistance profile, non-cytotoxicity to human cell lines, effectiveness in alleviating symptoms of specific diseases (e.g., colitis), ability to modulate gut microbiota composition, impact on host bile acid metabolism and signaling pathways (e.g., FXR-NLRP3), influence on inflammatory markers and cytokines	[144, 145]
*B. fragilis* ZY-312	Faces of a healthy infant	No	Facilitates colonic mucosa regeneration in colitis, motivates STAT3 signalling pathway, promotes IL-22 production, increases the percentage of IL-22-secreting type 3 innate lymphocytes (ILC3)	Alleviated DSS-induced colitis in mice, motivated STAT3 phosphorylation in colonic mucosa, promoted IL-22 production and increased ILC3 in colitis, facilitated colonic mucosa proliferation, mucus secretion, altered gut microbiota	DSS-induced colitis mouse model	This paper focused on the beneficial effects of *B. fragilis* strain ZY-312 in colitis models. However, [24] employed a comprehensive safety evaluation for the probiotic candidate Bacteroides fragilis strain ZY-312, demonstrating its safety through: 1) confirming typical characteristics of *B. fragilis*; 2) establishing its identity with 99.99% similarity to reference strain ATCC 25285 via whole genome sequencing; 3) identifying it as non-toxigenic without the bft gene; 4) verifying no significant adverse effects in both normal and immune-deficient mice; 5) demonstrating no risk of antibiotic resistance gene transfer; and 6) confirming its genetic stability over 100 generations of in vitro subculturing. These results robustly support ZY-312's suitability for probiotic applications	[177]
*B. xylanisolvens* AY11-1	Fresh faecal human samples	CP120351.1	Alleviating body weight loss, reducing contraction of colon length, reducing incidences of bleeding, attenuating mucosal damage in mice, improving gut dysbiosis, promoting the growth of probiotic bacteria including *Blautia* spp. and Prevotellaceae UCG-001 in diseased mice	Best capability for alginate degradation, production of significant amounts of oligosaccharides and short-chain fatty acids upon alginate degradation and fermentation, and amelioration of ulcerative colitis symptoms in dextran sulfate sodium (DSS)-fed mice	DSS-induced ulcerative colitis model in C57BL/6J mice	*B. xylanisolvens* AY11-1 was well-tolerated and demonstrated a good safety profile in both male and female mice, even at dosages up to 10 times the therapeutic level, underscoring its potential as a safe, next-generation probiotic	[49]
*B. fragilis* ATCC25285 (ATCC25285;DSM 2151)	American Type Culture Collection (Manassas, VI, United States), other source is not detailed	CR626927.1	upregulation of immune-related genes (CCN3, HAS2, and RICTOR), downregulation of inflammatory genes (EDNRB, TOX, and NKX2-3), enrichment in the regulation of the inflammatory response, cellular metabolism, and synaptic response pathways	Differential expression of genes related to immunity and inflammation, changes in the vaginal metabolomic profile particularly related to lipid metabolism and biosynthesis	Green shell laying chickens	-	[24]
*B. fragilis* NCTC 9343 (ATCC25285;DSM 2151)	American Type Culture Collection (Manassas, VI, United States), other source is not detailed	CR626927.1	Decrease *Salmonella* virulence in mice model, decrease in inflammatory cytokines and neutrophils infiltration, reduction of the genus Alistipes which could be related to the anti-inflammatory effects, bioactive fractions did not alter the gut microbiota diversity in mice	Only two fractions (F3 and F4) strongly inhibited S. Heidelberg translocation in a model mimicking the intestinal epithelium, reduction of S. Heidelberg in Peyer’s patches and spleen, downregulation of fliC expression	BALB/c mice	-	[54]
*B. plebeius* GDMCC 17135	Seaweed-eating humans	No	Inhibits muscle wasting in CKD, significantly decreases the levels of IL-1β, Il-6, and LPS in the serum, reduces skeletal muscle atrophy through the Mystn/ActRIIB/SMAD2 pathway, improves intestinal mucosal barrier function, restores gut microbiota homeostasis	Significant inhibition of muscle wasting in CKD, changes in the gut microbiome resulting in a significant reduction in gut permeability, reduction of serum endotoxin (LPS) levels, decrease in pro-inflammatory cytokines (IL-1β, IL-6)	5/6 Nx rats	-	[119]
*B. thetaiotaomicron* VPI-5482 (DSM 2079)	Human faeces, stored in DSMZ (Leibniz Institute DSMZ-German Collection of Microorganisms and Cell Cultures)	NC_004663	Restoration of intestinal homeostasis, amelioration of experimental ALD, preservation of mitochondrial fitness, redox state improvement	Decrease number in ethanol-fed mice, inhibition of growth by ethanol supplementation, restoration of alcohol-induced depletion, reduction of hepatic steatosis and lipid accumulation, partial restoration of mucus layer, increase of muc2 production, reduction of expression of cldn1 and MMP9, ERK phosphorylation, Hes1 expression	Lieber-DeCarli diet model of mice	-	[133]
*B. dorei* XR 2020	Faeces of a healthy human	No	Improves survival, reduces viral load, rapidly up-regulates IFNs expression in the lungs, enhances Th1 cytokine responses, suppresses Th2-mediated immune response, restores beneficial gut microbiota composition	Ability to metabolize quercetin, alteration of the gut microbiota composition, immunomodulatory effects through cytokine modulation, enhancement of type I interferon signaling, reduction of influenza virus replication in lungs	Influenza A virus A/Puerto Rico/8/34 mouse-lung-adaptive strain (PR8) in mice	-	[140]
*B. vulgatus* FTJS7K1	Faeces of a healthy human	JACBPY000000000.1	Alleviating lipopolysaccharide (LPS)-induced acute inflammation and intestinal injury, increasing the concentration of SCFAs, maintaining intestinal barrier integrity, protecting the integrity of intestinal epithelial cells	ability to alleviate LPS-induced inflammatory cell infiltration and goblet cells depletion, ability to maintain intestinal barrier integrity, presence of specific genes allowing adaptation to the survival pressure in the intestinal tract and mediating anti-inflammatory actions, influence on the concentration of SCFAs	6-week-old male SPF C57BL/6J mice	-	[159]

## Conclusion

The success of faecal microbiota transplantation in treating *Clostridium difficile*-associated diarrhoea highlights the potential of using specific commensal bacteria to address gut microbiota imbalances. *Bacteroides* spp., in particular, have emerged as promising candidates for treating a range of conditions, including intestinal inflammation, immune dysfunctions, metabolic disorders, and cancer prevention (see Table 2). The development process for these beneficial strains involves meticulous isolation, functional exploration, and rigorous safety assessments, following FDA and EFSA guidelines to ensure their safety for human use. This approach underscores the significant role next-generation probiotics could play in personalized medicine, leveraging the intricate relationship between the human body and its microbial inhabitants. In this respect here is an urgent need to sequence and annotate the genomes of potential isolates, enabling the prediction of their probiotic capabilities based on genomic content. This approach could significantly streamline the identification and utilization of new isolates with beneficial properties.

## Data Availability

No datasets were generated or analysed during the current study.
